# Bacteriophages as Therapeutic and Diagnostic Vehicles in Cancer

**DOI:** 10.3390/ph14020161

**Published:** 2021-02-17

**Authors:** Valentina Foglizzo, Serena Marchiò

**Affiliations:** 1 Department of Oncology, University of Torino, 10060 Candiolo, Italy; valentina.foglizzo@unito.it; 2 Candiolo Cancer Institute, FPO-IRCCS, 10060 Candiolo, Italy

**Keywords:** biopanning, drug delivery, gene therapy, phage display, targeting

## Abstract

Evolution of nanomedicine is the re-design of synthetic and biological carriers to implement novel theranostic platforms. In recent years, bacteriophage research favors this process, which has opened up new roads in drug and gene delivery studies. By displaying antibodies, peptides, or proteins on the surface of different bacteriophages through the phage display technique, it is now possible to unravel specific molecular determinants of both cancer cells and tumor-associated microenvironmental molecules. Downstream applications are manifold, with peptides being employed most of the times to functionalize drug carriers and improve their therapeutic index. Bacteriophages themselves were proven, in this scenario, to be good carriers for imaging molecules and therapeutics as well. Moreover, manipulation of their genetic material to stably vehiculate suicide genes within cancer cells substantially changed perspectives in gene therapy. In this review, we provide examples of how amenable phages can be used as anticancer agents, especially because their systemic administration is possible. We also provide some insights into how their immunogenic profile can be modulated and exploited in immuno-oncology for vaccine production.

## 1. Introduction

Cancer is a plague worldwide, affecting around 9.6 million people annually and accounting for 1 in every 6 deaths, globally. Adopting a healthy lifestyle lowers the risk of cancer development by 30–50%. Additionally, people suffering from cancer can more reliably count on preventive screenings, early detection, and access to affordable medicines (font: https://www.who.int/health-topics/cancer (accessed on 15 January 2021)). We live in the era of personalized medicine, where therapeutic interventions are built around the individual and are based on genetic screenings as well as on novel imaging techniques. The use of radiolabeled antibodies in Her2-positive breast carcinomas, for example, improved the detection of previously unidentified lesions, while genetic information rendered staging the disease more precise and led to the development of specific therapies [1,2].

Since the concept of targeted therapeutics came in vogue, efforts were made to gain a better understanding of tumor biology and to identify tumor-specific therapeutic targets. In fact, most of these targets are also predictive biomarkers that give us information on whether a certain therapy would be beneficial to one patient but not to another [3]. Furthermore, acquiring a deep understanding of the signaling pathways in which critical targets are involved is fundamental to dig out molecular mechanisms that will likely inform us on response—or resistance—to therapy [4]. Whether a tumor would respond or not is also a matter of selective drug accumulation [5]. Therefore, there is a necessity for a more careful drug design following the principles of low toxicity, targeting capacity, and high payload at the disease site [6]. The same is true for diagnostic agents that should necessarily biodistribute rapidly, accumulate specifically, and cause no harm to the individual [1]. Therefore, more drugs are being designed on the defined features of cancerous tissue to avoid unwanted side effects. It is in fact unconceivable to solely screen new compounds for their activity on specific cellular processes, as their effectiveness is often impaired by unbearable off-target toxicities.

To fulfill the need of more precise diagnostic and therapeutic medicines, a multitude of new strategies were implemented. Among those, the advent of nanotechnology opened up new roads in precision medicine with nanocarriers or nanoparticles (NPs) made up of different materials (namely, magnetic, iron oxide, gold, mesoporous silica, polymeric NPs, carbon nanostructures, and liposomes) showing good biocompatibility, relatively low toxicity, and simple customization to reach the defined parameters, such as good circulation lifespan and solubility [6]. With formulations already approved for the treatment of metastatic breast cancer (pegylated liposomal doxorubicin Doxil^®^; non-pegylated liposomal doxorubicin Myocet^®^) and others in late stages of clinical trial for different solid tumors, liposomes obtained the greatest success, especially for their low immunogenicity and toxicity [7]. All these nanocarriers, however, lack intrinsic target specificity and accumulate at the tumor site because of its leaky vasculature and the so-called enhanced permeability and retention (EPR) effect that causes greater fluid retention than in normal tissues [8,9]. In addition, they have to bypass a number of physiological barriers, namely the endothelium, the extracellular matrix (ECM) and different stromal cells, including phagocytic immune cells, before releasing their cargo at the target site [10,11].

It is only in recent years that scientists started to develop strategies to target synthetic and biological nanocarriers to a desired cell; most of this work is inspired by bacteriophage research. This review focuses on how bacteriophages are employed in oncology to build a new class of intelligent drugs with the unique ability of targeting tumor tissues, while sparing their normal counterpart. In particular, the review focuses on (i) phages with a better capacity of bringing a high payload of therapeutics and diagnostics to cells, (ii) phages that are vehicles for DNA and RNA delivery in gene therapy, and (iii) phages that act as vaccine carriers.

## 2. Bacteriophages: An Overview

Bacteriophages are a class of prokaryotic viruses with different sizes and shapes (icosahedral, such as T4, T7, or λ; filamentous, such as M13), which infect the host bacterial cell initiating either a lytic or a lysogenic cycle. Lytic phages (e.g., T4) replicate inside the bacterial cell and produce endolysins to destroy the cell membrane and release the viral particles. Lysogenic phages (e.g., λ and T7), instead, integrate their genes into the bacterial genome (prophage) and are transmitted to the next generations. Under conditions such as stress or cellular damage, the prophage can be reactivated, viral particles can be produced, and a lytic cycle can be initiated [12]. Filamentous phages always behave as lysogenic viruses and never kill the host cell. Their genome can either be replicated as episomal DNA or integrated into the bacterial chromosome by recombination. In the latter case, reactivation of viral replication is achieved under certain stimuli (modification of pH or temperature) that also promote reassembly of new viral particles [13,14]. In the infection process, bacteriophages and host cells establish irreversible interactions that take place between the viral capsid proteins and specific receptors on the bacterial surface. In filamentous phages, contact with the host cell is mediated by the minor capsid protein that binds the bacterial F-Pilus. Pili retract towards the bacterial cell and promote the interaction of the phage with a secondary receptor located in the periplasm. With mechanisms that are still not fully understood, the major coat protein is inserted into the inner membrane and the phage ssDNA is released inside the cell [13,14]. In tailed icosahedral bacteriophages, upon binding through the tail fibers, a conformational change is induced in the capsid baseplate, which ultimately leads to capsid contraction and injection of phage’s genetic material (ds or ssDNA/RNA) into the bacterial cytoplasm [15].

Bacteriophages were discovered in 1915 and 1917 by Frederick Twort and Felix d’Herelle, respectively, for their use against human and animal infections, but research on the theme was abandoned with the advent of common antibiotics. They are applied as food preservatives, as surface biofilm degradants, and in many other applications that are extensively reviewed elsewhere [15,16]. It was only in 1985 that bacteriophage research for human therapy started to get off the ground. With the phage display technique, in fact, bacteriophages transformed into a platform to study protein, antibody, and peptide interactions. Bacteriophages can be genetically modified so that a polypeptide is produced and exposed as fusion with one of their capsid proteins. The different capsid proteins within a single phage particle are present in numbers that might vary from a handful to several hundreds. Thus, the surface of a phage can be variably decorated with multiple copies of the polypeptide of interest and within a phage population, a multiplicity of moieties are rapidly and easily displayed to create a library [17,18]. Libraries are propagated in bacteria and phage populations can be selected either in vitro or in vivo by biopanning—an affinity procedure that makes interacting phages to be retained while non-interacting phages are washed away. Biopanning on cell cultures, for example, is a straightforward method to identify cell surface-interacting peptides and to uncover novel tumor-associated antigens for the design of targeted delivery systems [11]. In recent years, bacteriophage research entered its second youth, with phages being exploited either in cancer therapy and diagnosis as targeted nanocarriers or employed in gene therapy as vehicles for curative DNAs/RNAs. Furthermore, phages as immunogenic particles are described in vaccine research for their capability to elicit both cell-mediated and antibody-mediated (humoral) immune responses [19].

## 3. Discovery of Cancer Biomarkers and Targeting Moieties by Phage Display

Smith and Winter paved the way for bacteriophage engineering and received the Nobel Prize in chemistry in 2018 for their contribution in establishing phage display [20]. This technique is based on the possibility of creating fusion proteins with antibodies or peptides, by cloning the sequence of interest at the N- or C-terminus of a capsid protein. The filamentous phage M13 is the most widely and successfully exploited. In this phage, exogenous peptides are expressed as a fusion with either the major coat protein gp8 or the minor coat protein gp3. The difference relies on the number of peptides with which the capsid surface is decorated, where the gp3 is positioned at one end of the bacteriophage and is expressed in 5 copies, while the gp8 protein covers the entire length of the phage and is expressed in ~2700 copies [21]. As a result, engineered phages have the ability to recognize and bind, by affinity and avidity, receptor molecules on the surface of a cell and interfere with their function [22]. Other bacteriophages used in phage display include the icosahedral phages λ [23], MS2 [24], T4 [25], and T7 [26]. All these platforms share the same biotechnological strategy, i.e., variable copies of exogenous peptides/antibodies are displayed in different copy numbers as fusion with one or more capsid proteins (Table 1).

Phage display is a high-throughput technology to select protein moieties (namely, peptides, proteins, antibodies, or single-chain variable fragments (scFvs)) starting from complex libraries, with a multiplicity that can reach 10^6^–10^9^ individual clones. The affinity selection is carried out in vitro or in vivo, based on the interaction of the library with a target of interest—a protein, a cell, a tissue, an organ, or even a whole organism. Sequential rounds of selection and amplification, followed by recovery of specifically enriched phage clones, enable the identification of previously uncharacterized moieties and their molecular targets. In the last 30 years, several studies sought to identify cancer-specific biomarkers and cognate targeting peptides/antibodies, through the use of phage platforms for various downstream purposes. There is a vast literature on the subject, whose full discussion goes beyond the scope of the present review; here, we propose an overview of different possible approaches, to introduce the reader to the multifaceted applications of phage display in oncology.

Poul et al. [31] generated an scFv phage library to select internalizing antibodies as a prerequisite to design targeted therapeutics (e.g., immunotoxins, immunoliposomes, antibody–drug conjugates) and gene delivery systems. They built a library of 7.0 × 10^9^ human scFvs in an M13-derived phagemid, to be selected on the breast cancer cell line SKBR3. Depletion of unspecific binders was obtained on normal cells (fibroblasts) and recovery of SKBR3-internalizing scFvs was achieved after three rounds of panning. Out of 135 clones tested, they found two scFvs (clones F5 and C1) that uniquely bound to Her2, a receptor frequently overexpressed in breast cancer. For further characterization, they showed that in between the non-Her2 binders, 10 other scFvs were able to specifically interact with the cancer cell lines of different origin (breast, SKBR3 and MCF-7, ovary, SKOV3, and prostate, LNCaP). They retrieved one clone to selectively bind transferrin (Tf) receptor—another cell surface molecule often upregulated in cancer—and to induce, upon internalization, a strong growth inhibitory effect. Similarly, Tordsson et al. [32] employed phage display to uncover novel target molecules for immunotherapy and diagnosis of colon cancer. In this case, an M13 gp3-fused library of 2.7 × 10^7^ scFv clones was derived from antibodies induced in Cynomolgus monkeys, upon immunization with human colon carcinoma cells. The library was panned on the human colorectal cancer cell line Colo205 and the selected clones were used to stain tissue sections of normal and cancerous tissues. This procedure allowed the identification of an scFv clone (A3), specific to both primary and metastatic colorectal cancer and also to pancreatic cancer, with restricted cross-reactivity to normal epithelia.

Using a different setting, Pavoni et al. [33] implemented an immunological screening protocol called SEREX, where a λ phage library was constructed by expression of cDNA-encoded protein fragments (200–300 amino acids in length) from the breast cancer cell lines MCF-7 and MDA-MB-468. This library was panned on sera from breast carcinoma patients, where circulating antibodies against cancer antigens are expected to be present in high titers. Twenty-one clones that specifically interacted with the sera were retrieved and their sequence were determined. Eighteen different gene products were found and their expression pattern was validated by semi-quantitative RT–PCR in 10 independent breast carcinoma samples, leading to the identification of five antigens (T6-2, T6-7, T7-1, T9-21, and T9-27) associated with cancer diagnosis.

An example of phage display performed on whole tissues is the work by Larsen et al. [34]. They screened an M13-displayed synthetic single domain antibody library [35] with a diversity of 6.2 × 10^7^ on consecutive tissue sections for binding to CD271+ cells, considered as potential cancer stem cells (namely, cells that fuel tumor growth and are often drug-resistant). Of the 315 screened, they validated one clone (LH8) through immunohistochemical staining on cryo-preserved breast cancer tissue sections. This antibody showed confined reactivity to cancer cells with variable intensities within the tissue, confirming an intratumor heterogeneity of different cell populations and indicating a possible application in the precise identification of cancer stem cells.

Other studies used recombinant proteins as targets. Mueller et al. [36] searched for peptides that could bind to collagen type IV, specifically cleaved by the matrix metalloprotease 2 (MMP-2), an event that occurs during angiogenesis—the abnormal formation of new blood vessels that feed tumor growth. They found one M13 phage clone exposing the peptide motif TLTYTWS to bind immobilized MMP-2 processed collagen IV. The TLTYTWS peptide inhibited endothelial cell differentiation in a tube formation assay in vitro, blocked angiogenesis in Matrigel plug assays in vivo, and accumulated at tumor sites in Lewis Lung carcinoma-bearing mice. A work by Zhang et al. [37] focuses on CD44, a member of the family of integral membrane glycoproteins and the receptor for hyaluronic acid. CD44 is often aberrantly expressed in cancer and it is also associated with epithelial-to-mesenchymal transition, a crucial event in cancer progression. Consequently, generating new diagnostic tools for CD44 can lead to more efficacious treatments and predict response to therapy. Based on these premises, the authors searched for novel peptide ligands of CD44v—a splice variant often deregulated in gastric cancer—starting from a commercial M13 phage library of 1 × 10^9^ independent 7-mers. They identified CV-1, the phage clone with the highest frequency and selectivity, to specifically bind the gastric cancer cell line SGC-7901, and stain both gastric cancer tissue and metastatic lymph nodes. Recently, Zuo et al. [38] sought to inhibit neovascularization mediated by vascular endothelial growth factor (VEGF) and its receptor VEGFR2. They generated a mini-library of T4 phages expressing different fragments of the extracellular domain of VEGFR2 fused with the Soc capsid protein, showing that all these fragments interacted equally well with plate-immobilized VEGF. Adding T4-VEGFR2 phages to cultured endothelial cells, significantly impaired their proliferation and migration through inhibition of VEGFR2 phosphorylation and downstream signaling. Intravenous administration of T4-VEGFR2 phages in mouse xenografts of Lewis lung cancer and colon cancer, greatly inhibited tumor growth, micro-vessel density, and tumor vascularization, in general.

## 4. Bacteriophages and Their Derivatives for Drug Delivery and Targeted Imaging

Phage display-identified peptides are extensively used in tumor-targeting approaches. Peptides penetrate easily within a given cell or tissue due to their relative small size. Furthermore, they can be rapidly conjugated to any drug or carrier with a simple and cheap chemistry. Thus, peptides, within protein-based nanocarriers, represent the driving force to identify target molecules and transform new generation drugs into self-navigating formulations [39]. So far evidence shows that conjugation with tumor-homing peptides ameliorates the therapeutic index of conventional chemotherapeutics, by lowering the side effects and allowing higher drug accumulation at the disease site. Applications are multiple and were extensively reviewed elsewhere [6]. Here, we report a few examples of peculiar studies on this subject.

Shadidi and Sioud [40] employed M13-based 7- and 12-mer commercial phage libraries to screen for breast cancer-specific internalizing peptides. Internalization is a feature of some peptides that upon recognition with surface receptors penetrate within the cell via bona fide endocytosis/macropinocytosis [41], thus, shuttling a conjugated active agent into the cytoplasm. After 5–6 rounds of panning on the Her2-positive SKBR3 breast cancer cell line, the authors found two peptide motifs, LTVSPWY, and WNLPWYYSVSPT, specific for breast cancer cells, the latter was also capable of binding to glioma, prostate, colon, and lung cancer cells. Both peptides were shown to internalize and deliver fluorescein-conjugated anti-Her2 antisense oligonucleotides into SKBR3 cells. Du et al. [42] employed a 12-mer commercial M13-displayed library for in vivo biopanning of xenograft models of BEL-7402 hepatocarcinoma (HCC). In these experiments, phages were injected intravenously, followed by animal killing after a short circulation time (15 min), and recovery of specific clones from explanted tumor masses. After testing the 130 tumor-enriched phage clones, they selected the clone A54 displaying the motif AGKGTPSLETTP for further validation. Doxorubicin (Dox) conjugated to the A54 peptide exerted a strong antitumor activity in HCC tumor-bearing mice, improving the overall survival and causing no severe side effects, as compared to the unconjugated drug. A strategy to target tumor angiogenesis was developed by Fukuta et al. [43]. They employed sequential in vitro and in vivo biopanning of random pentapeptides fused with the minor coat protein gp3 of M13 filamentous phage. The library was first panned on cultured human endothelial progenitor cells (hEPCs), followed by in vivo panning of the recovered phages in the dorsal air sac (DAS) model, in which a chamber ring loaded with B16 melanoma cells is dorsally implanted in mice. Among the phages recovered from the newly formed vessels around the ring, a clone displaying the ASSHN motif was found to be the most frequent and was validated in vitro and in vivo for binding to both hEPCs and the neo-vessels, respectively. The authors showed that this peptide homes to the vessels and leaks through them into the tumor. Upon conjugation with Dox-loaded liposomes, ASSHN distributed into the tumor and drove Dox-dependent growth inhibition to a much greater extent, as compared to untargeted liposomes. An interesting evolution of these applications combines liposomes and phage-derived proteins to target Dox in a model of human breast cancer. In a series of studies by Torchilin’s group, a tumor-targeting 8-mer was first selected by panning an M13-based library displayed on the major coat protein gp8 [44]. The whole 55-amino acid fusion protein, including both gp8 and the cancer-targeting 8-mer, was purified and incorporated into the lipid bilayer of Doxil. This formulation, named MCF-7-targeted phage-Doxil, showed cytotoxic effect in vitro and induced a significant reduction in tumor growth, without affecting the body weight, when administered to MCF-7 xenograft-bearing mice [45].

Bacteriophages themselves are particles with therapeutic potential. They can be vehicles for curative nucleic acids, and decoration of their capsids with drugs and imaging dyes transforms phages into theranostic platforms. Original work by Cai et al. [46] showed that a plasmid coding for a siRNA against focal adhesion kinase (FAK) could be easily packed into M13 particles displaying epithelial growth factor (EGF) as a fusion with the gp3 protein. The deriving siRNA-carrying phages were efficiently targeted to the EGF receptor (EGFR)-overexpressing H1299 lung carcinoma cells, with consequent inhibition of cell growth and invasiveness in vitro. In another work, Huang et al. produced derivatives of HK97 phage by chemically crosslinking a labeling dye (fluorescein), a targeting moiety (Tf), or both, to the capsid protein [47]. The dual-functionalized viral nanoparticles (VNPs) incubated with Tf receptor-overexpressing tumor cells were internalized and targeted to the endolysosomes, suggesting that they might be amenable for imaging and delivery of therapeutic molecules in vivo. Similarly, M13 phages targeted to prostate cancer cells via an anti-prostate-specific membrane antigen (PSMA) antibody were successfully employed as imaging agents. In this system, the antibody was fused with the gp3 protein, while near infrared (NIR)-fluorescent single-walled carbon nanotubes (SWNTs) were assembled through a peptide ligand fused with the gp8 protein. The deriving anti PSMA-M13-SWNT, when injected in tumor-bearing mice showed targeted uptake, thus, holding great promise for in vivo fluorescence imaging [48].

New therapeutic applications in oncology include photodynamic therapy in which light and a photosensitizer react together to form reactive oxygen species that kill cancer cells. Usually, the major side effects come from accumulation of the photosensitizer in off-target tissues and its uneven spreading caused by the EPR effect. To overcome this issue, Cohen et al. [49] employed MS2 bacteriophages decorated with nucleolin-targeting DNA aptamers to deliver the photosensitizer meso-tetra-(4-*N*,*N*,*N*,-trimethylani-linium)-porphine (TMAP) to breast cancer cells in vitro. In this experimental setting, the photosensitizer was incorporated into the phage capsid during the assembly process, while the aptamer was chemically crosslinked to the phage surface. This study showed that an equivalent of 2–3 μM TMAP administration was able to reduce cancer cell viability by 50%. Similarly, M13 bacteriophages targeted to SKBR3 breast cancer cells via a gp8-fused peptide (of sequence VSSTQDFP) and carrying the photosensitizer pyropheophorbid-a induced specific cancer cell death, as compared to untargeted phages [50].

In another line of research, enzyme replacement therapy or VNP-mediated enzyme prodrug therapy was envisaged as a new treatment modality against cancer. Sánchez–Sánchez et al. [51] expressed CYPBM3, a variant of Cytochrome P450 from *Bacillus magaterium*, as a fusion with the capsid protein of bacteriophage P22. The deriving P22-CYP VNPs load a higher amount of enzyme compared to other nanocarriers and deliver it to the cells that are almost completely active. Although a functionalization of the VNPs with a targeting ligand for mammalian uptake was not made in this work, the authors comment on its feasibility as a way of delivering an enzyme that efficiently converts a prodrug in the tissue of choice.

**Figure 1:**
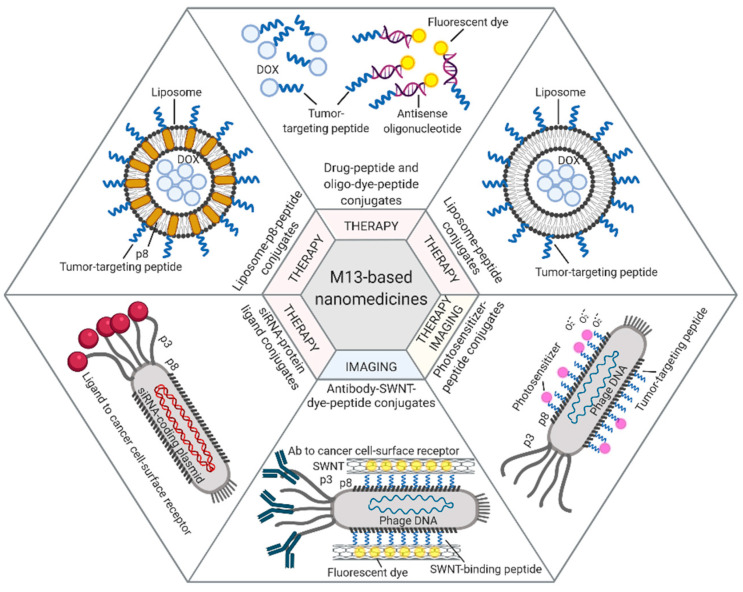
M13-based applications. Examples of therapeutic and diagnostic approaches exploiting M13 phage display-derived peptides [40,42], capsid proteins [44,45], and whole phages [46,48,50] as carriers for tumor-targeted delivery of drugs (e.g., Dox, antisense oligonucleotides, photosensitizers) or imaging dyes (e.g., fluorescein, NIR-SWNTs). Figure created with BioRender.com.

Among the different approaches described in this paragraph, prototype applications based on the M13 bacteriophage are summarized in Figure 1.

## 5. Bacteriophages and Their Derivatives for Gene Therapy

Gene therapy requires safe and efficient delivery systems that can target a specific cell or tissue. Bacteriophage-mediated gene therapy is now possible, due to their adaptability. Transgene delivery typically employs eukaryotic viruses, as they transduce cells with high efficiency. However, their native tropism for mammalian cells severely limits their use, as it leads to unwanted side effects. Bacteriophages represent a safer option as they can be re-engineered to transduce specific eukaryotic cells or tissues.

**Figure 2:**
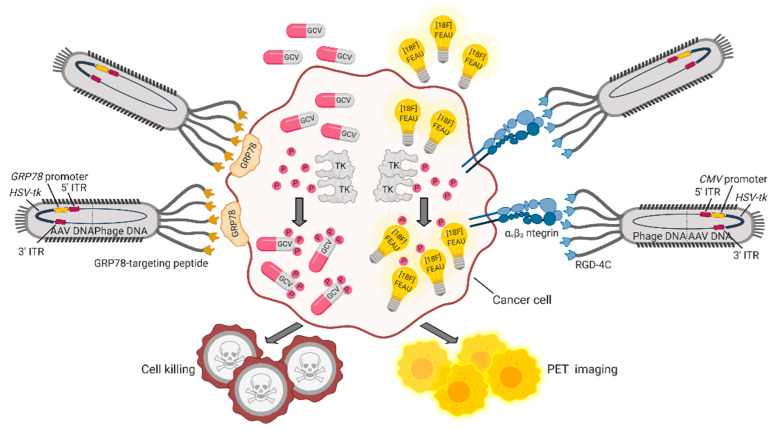
Theranostic applications of the hybrid AAVP vector. AAPVs can be ligand-targeted to a tumor-specific cell surface molecule such as α_v_β_3_ integrin [53] or GRP78 [59], followed by internalization of DNA and production of AAVP-coded proteins. The *HSVtk* transgene codes for TK, an enzyme that adds phosphate groups to thymidine analogues and converts (i) the prodrug GCV in the cytotoxic drug GCV triphosphate and (ii) [^18^F]FEAU in [^18^F]FEAU phosphate, which is retained intracellularly, allowing detection by PET imaging. Figure created with BioRender.com.

The double cyclic RGD peptide (CDCRGDCFC, RGD4C) that targets αvβ3 integrin, a marker of cancer vasculature and tumor tissue, was successfully exploited as a targeting moiety (by the research group of Pasqualini and Arap) to build up a hybrid adeno-associated virus (AAV)/M13 phage (P) system termed AAVP. This vector carries an eukaryotic AAV cassette in the intergenomic region of the RGD4C phage, which is packaged within the viral capsid, together with phage DNA [52]. An AAVP vector engineered to include the *GFP* gene under the *CMV* promoter was shown to be efficiently internalized by cancer cells. When systemically administered to human Kaposi sarcoma xenografted mice, RGD4C-AAVP accumulated in tumor vasculature by a ligand-directed mechanism. Similarly, an AAVP modified with a luciferase reporter gene for bioluminescence imaging showed specific accumulation in tumors and no accumulation in normal organs. To potentially translate this system into the clinic, a Herpes simplex virus-1/thymidine kinase (*HSVtk*) expression cassette was introduced to be exploited (i) as a suicide gene upon administration of ganciclovir (GCV) and (ii) as a positron emission tomography (PET) imaging platform, in the presence of the radiolabeled nucleoside analog 20-[^18^F]-fluoro-20-deoxy-1-β-D-arabino-furanosyl-5-ethyluracil ([^18^F]FEAU). With this system, tumor response to GCV treatment was monitored across cohorts of human-cancer-cell-xenotransplanted mice showing reduction of tumor volume and destruction of tumor vasculature at histopathological analysis. In another work by the same group, a similar approach was used to treat and image nude rats bearing human sarcoma tumors with the purpose of creating a preclinical model to predict drug response, when the overall tumor-size change does not represent a good read-out of therapy outcome [53]. An implementation of these AAVPs was designed by including an alternative promoter and a different targeting moiety. Glucose-regulated protein 78 (GRP78) is an endoplasmic reticulum protein chaperone, whose expression is induced in a variety of tumors, in conditions of glucose deprivation and hypoxia. Therefore, the *GRP78* promoter, besides being specifically activated in cancer cells, is expected to improve gene expression compared to the *CMV* promoter, also because it is not silenced in eukaryotic cells [54]. Of note, upon a single systemic administration of either AAVP-RGD4C/*GRP78*-*HSVtk* or AAVP-RGD4C/*CMV*-*HSVtk* in L9 gliosarcoma-xenotransplanted nude mice, followed by GCV treatment, tumors showed similar responses. However, repeated administration of GCV in large tumors showed efficacy only in AAVP-RGD4C/*GRP78*-*HSVtk* treated mice, further confirming transcriptional delivery of the transgene in a stress-permeated setting [54]. A number of other promoters were assessed in vitro and in vivo to optimize the delivery efficiency of suicide genes, suggesting that some of them—namely, carcinoembryonic antigen (*CEA*) [55,56], epithelial cell adhesion molecule (*EpCAM*) [57], human telomerase reverse transcriptase (*hTERT*) [58]—might as well be suitably included in AAVP for cancer targeting. In several cancer settings, the GRP78 protein is not only overexpressed, but is also relocated from the cytoplasm to the cell surface, becoming suitable for ligand-targeted approaches. For example, moderate to strong levels of GRP78 are seen in inflammatory breast cancer (IBC), an aggressive disease characterized by difficult-to-detect micrometastasis at presentation. AAVPs displaying the GRP78-targeting peptide motif WIFPWIQL were employed to image IBC tumors in vivo with NIR-conjugated dyes. The positive outcome of these preliminary studies was translated into a theranostic platform to deliver the *HSVtk* suicide gene via a GRP78-targeted AAVP carrying the *GRP78* promoter. Results demonstrated improved imaging and decreased tumor size upon GCV administration, as a result of ligand-directed accumulation of the processed drug within the tumor [59]. A schematic representation of these theranostic AAVP vectors is provided in Figure 2.

Well-known, biologically active peptides are also a source of targeting ligands that do not require a priori screening. For example, octreotide (OCT), a synthetic analog of somatostatin, was exposed on AAVP for the delivery of tumor necrosis factor (TNF) to somatostatin receptor type-2 (SSTR-2) expressing pancreatic neuroendocrine tumors. Mid- and long-term systemic injection of OCT-AAVP-TNF in transgenic mice with pancreatic insulinomas induced tumor response, as determined by the decreased insulin secretion, total choline levels, and tumor sizes [60]. Transduction in AAVP systems remains a challenge due to physiological limitations in viral delivery and processing. In particular, both the ECM and the proteasome system pose a barrier to AAVP efficacy. Hence, ECM depletion by specific enzymes (collagenase and hyaluronidase) and proteasome inhibition by peptide aldehyde inhibitors (*N*-benzyloxycarbonyl-L-leucyl-L-leucyl-L-leucinal (MG132) and *N*-acetyl-L-leucyl-L-leucyl-leucyl-L-norleucinal (LLnL)) were shown to improve both viral uptake and transgene expression [61,62].

**Figure 3:**
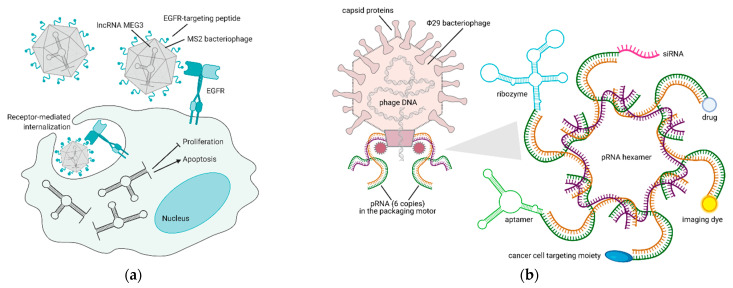
Bacteriophage-based platforms to deliver therapeutic and diagnostic agents via RNA technology. (**a**) Reintroduction of MEG3 lncRNA in cancer cells via an EGFR-targeted MS2 phage—upon binding to EGFR, the phage is internalized by endocytosis and releases MEG3, thus inducing apoptosis and inhibiting proliferation of cancer cells [63]. (**b**) VNPs derived from Φ29 bacteriophage pRNA are suitable nanocarriers for the delivery of RNAs (aptamer, ribozyme, siRNA), small molecules (drug, dye), or proteins (targeting moiety) to cancer cells [64,65]. Figure created with BioRender.com.

Bacteriophage-based VNPs are, at present, the only reliable alternative to liposome-mediated noncoding RNA delivery. Their stability at physiological and supraphysiological conditions proved them to be resistant even at acidic pH and high temperatures, and encapsulation within the viral capsid protects RNA molecules from degradation. Long-noncoding RNAs (lncRNAs) are cell regulatory RNAs, typically more than 200 nucleotides in length, whose expression is often lost in cancer. LncRNA MEG3 re-expression in HCC models is the only rescue attempt that was published so far. Within this publication, the EGFR-targeting peptide YHWYGYTPQNVI (named GE11) was crosslinked to the capsid of MS2 bacteriophage-derived VNPs, in order to achieve MEG3 endocytosis. MEG3 reintroduction was shown to cause growth arrest and apoptosis via p53 stabilization in vitro and to impair tumor growth in vivo [63] (Figure 3a).

As a peculiar yet promising application in phage-derived therapeutics, RNA nanotechnology offers the possibility of manipulating the DNA packaging unit of bacteriophage Φ29, to make chimeric RNAs that form dimers via the right and left-hand loop interactions. Within the bacteriophage, packaging RNA (pRNA) dimers serve as building blocks for bottom-up assembly of pRNA hexamers that compact its DNA in the procapsid. pRNA dimers are 25 nm in size and sequence replacement of their helical domain does not alter dimer formations. Thus, redesign of monomers to carry siRNAs for gene downregulation and aptamers/ribozymes for cell targeting is possible and is shown to produce dimers that mediate gene silencing in different cell types [66]. As an example, specific dimers of pRNA-folate and pRNA-siRNA survivin or metallothionein II were shown to degrade the corresponding mRNAs in ovarian cancer cells much more efficiently than siRNAs alone [67]. Furthermore, changing these assemblies to build modules of up to 12 monomers with different geometries was demonstrated to lead to stable RNA nanoparticles that can also be systemically administered [64]. Recently it was also shown that the 3-way core junction of Φ29 pRNA could be functionalized to carry two siRNAs and a targeting RNA aptamer. Specifically, Her2+ breast cancer cells were targeted with an RNA nanoparticle carrying two siRNAs for *MED1* (a well-known transcriptional co-activator of Her2) and an RNA aptamer targeting Her2. pRNA-Her2apt-siMED1 effectively silenced *MED1* in breast cancer cells, blocked tumor growth, and completely eradicated lung metastases [65] (Figure 3b). These results clearly point towards future application of these RNA-based nanoparticles in the clinic.

## 6. Bacteriophages as Carriers for Cancer Vaccines

Filamentous bacteriophages such as M13 are capable of inducing immunity at different levels, as excellently discussed in reviews by De Berardinis’ group [68,69] and others [70]. Being particulate, they are uptaken and processed by antigen processing cells (APCs), followed by presentation of the deriving peptides by the major histocompatibility complex (MHC) class I and class II molecules. As a consequence, both CD8+ and CD4+ T lymphocytes are activated, respectively, inducing a strong and complete cell-mediated response [71,72]. To optimize recognition by APCs, Sartorius et al. produced M13-derived bacteriophages displaying an scFv specific for DEC-205, a surface molecule expressed by dendritic cells, as fusion with the gp3 capsid protein [68]. Such APC-targeted phages induced maturation of dendritic cells in vitro and in vivo, with secretion of interferon α (IFN-α) and interleukin 6 (IL-6). The vaccine was further implemented by including the tumor-associated antigen OVA257-264 as fusion with the gp8 capsid protein. The deriving dual displaying phage (fdOVA/sc-αDEC) stimulated a strong CD8+ T cell response and delayed tumor growth in mice xenografted with melanoma B16-OVA cells.

Filamentous bacteriophages also trigger humoral immunity, even in the absence of added adjuvants [73,74]. In fact, the capsid proteins of M13 act as adjuvant themselves, as demonstrated in a pioneering work by Cuesta et al. [75]. In this study, the authors engineered a plasmid for the expression of a fusion between the gp3 protein of M13 and an scFv against the extracellular protein laminin-1. In vivo administration of this plasmid led to the production of specific antibodies and high amounts of IFN-λ, a cytokine secreted by activated T cells and natural killer cells. In contrast, administration of a plasmid coding for the anti-laminin-1 scFv only was unable to trigger an immune response. Similarly, the intrinsic antigenicity of filamentous phages can be exploited to overcome immune tolerance toward self-antigens. For example, Bartolacci et al. [76] sought to raise a vaccine against Δ16HER2, a splicing variant of Her2 with a role in aggressiveness and drug resistance of breast cancer. Δ16HER2 DNA vaccine administered to mice bearing Δ16HER2-expressing tumors did not provide immune protection. In contrast, a bacteriophage displaying Δ16HER2 epitopes fused with the gp3 capsid protein (pVAX-Δ16ECTM), and even one expressing epitopes of wild-type Her2 (pVAX-hECTM), efficiently induced protective immunity in a mouse model of breast cancer. As 50–92% of Her2+ breast cancers express the splice variant Δ16HER2, the approach might be a valuable starting point to develop next-generation vaccines.

In all these examples, M13-derived bacteriophages were genetically engineered to display tumor-associated antigens. An alternative is to chemically link the antigen onto the phage surface, as described by Roehnisch et al. [77]. In this study, antigenic antibody fragments expressed on the surface of malignant B cells (tumor-specific idiotypes) were produced as recombinant proteins, purified, and linked to the M13 major coat protein gp8. The chemically modified bacteriophage proved superior in inducing a humoral response, when compared to its genetically engineered counterpart and with the same antigen linked to a standard adjuvant (namely, keyhole limpet hemocyanine), probably due to the higher numbers of displayed antibody fragments.

Among other phage-based platforms, the icosahedral bacteriophage λ is extensively employed in vaccine design, because unlike M13, its genome is permissive to the insertion of quite large portions of exogenous DNA (up to 20 kb). The first applications used λ phages to deliver prototype DNA vaccines, in order to evaluate the potential advantages of this approach over naked DNA. In these studies, the DNAs coding for different proteins—GFP, hepatitis B surface antigen (V-HBsAg), and other antigens derived from genome libraries of animal pathogens—were individually cloned in the λ-gt11 vector, under the control of a *CMV* promoter. As expected, the deriving phages were efficiently processed by APCs, and regardless of the antigen carried, they induced higher titers of specific IgGs than plasmid DNA [78]. A successive work from the same group redefined the doses and administration schedules of the λ-V-HBsAg vaccine in both mice and rabbits (as an example of larger animal), confirming previous results on its efficacy and paving the way for more detailed dosage/pharmacokinetics analyses [79]. Similar approaches were adopted to engineer λ bacteriophages in the design of anticancer vaccines. Ghaemi et al. [80,81] investigated the feasibility of a therapeutic vaccine against cervical cancer. For this purpose, they inserted the gene coding for human papilloma virus 16 (HPV16) protein E7 in a Lambda ZAP^®^ CMV vector, obtaining the λ-ZAP HPV16 E7 bacteriophage. The efficacy of this vector in inducing an immune response was first evaluated ex vivo on cells from immunized mice, showing higher cytotoxic activity, increased proliferation upon antigen stimulation, increased release of granzyme B (involved in cytotoxic responses and antiviral defense) and IFN-γ, compared to the control. In vivo, λ-ZAP HPV16 E7 bacteriophage delayed the growth of xenografted E7-expressing tumors in the TC-1 mouse model. This was the first study demonstrating the anticancer potential of λ-based vaccines. Despite a relative success of DNA-based (genetic) vaccination approaches, displaying peptide/protein epitopes as fusion with the gpD capsid proteins of λ bacteriophage is more effective in the activation of both humoral and cell-mediated immunity, as demonstrated in a comparative analysis by Thomas et al. [82]. On these bases, recently, Behravan’s group engineered λ bacteriophages to express different epitopes of Her2, namely E75 (corresponding to Her2 amino acids 369-377) [83], AE37 (776-790) [84], and GP2 (654-662) [85], as a fusion with the capsid protein gpD. These vaccines showed different induction of immune responses, the AE37 and GP2 epitopes being superior in mediating both preventive and curative effects in mice xenotransplanted with TUBO breast cancer cells, accompanied by a strong activation of cytotoxic T lymphocytes.

Finally, the T4 and T7 bacteriophages were also used in pilot experiments as potential vaccine carriers. Work by Ren et al. describes the engineering of T4 to express murine VEGFR3/Flt4 (T4-mFlt4), as a fusion with the capsid protein Soc [86]. Flt4 is a transmembrane receptor involved in tumor lymphangiogenesis, with a role in tumor growth and metastasis, therefore a vaccine against this protein is expected to have a protective role against tumor progression. T4-Flt4 was evaluated in the Lewis lung carcinoma mouse model by a schedule of prophylactic vaccination. Although this treatment did not have any effect on the engraftment and growth of xenografted tumors, the vaccine was capable of inducing specific antibodies and inhibiting both lymphangiogenesis and distant metastasis. In a similar setting, the same authors produced a T4 phage displaying murine VEGFR2 (mVEGFR2) [87], which was tested in the same animal model. In this case, the T4-mVEGFR2 phage significantly delayed tumor growth in vaccinated mice and triggered the production of antibodies with neutralizing properties, as confirmed in different in vitro angiogenesis assays. Adoptive transfer of these IgGs to Lewis lung carcinoma xenotransplanted mice, led to the inhibition of angiogenesis, delayed tumor growth, and extended survival. Pouyanfard et al. produced a T7 phage exposing a nonapeptide of rat Her2 named p66, which corresponded to amino acids 66–74 and was identified as a H-2K^d^-restricted, dominant cytotoxic T lymphocyte epitope [88]. They formulated two different constructs, displaying either the single epitope or a tandem repetition, named T7-p66 or T7-p66x2, respectively, and tested the potential efficacy of the deriving vaccines in the TUBO model of breast carcinoma. In these prevention experiments, cancer cells were significantly rejected by mice vaccinated with T7-p66 (but not T7-p66x2), leading to an overall survival >80% at the end of the observation period (42 weeks). These experiments also demonstrated that a correct exposition of the antigen is crucial to achieve an immune response. Recently, Shukla et al. engineered T7 bacteriophages to express short peptide fragments of 8 mutated proteins (murine gene codes, *Fzd7*, *Atp11a*, *Dag1*, *Gnas*, *Atrn*, *Pcdhga11*, *Sema3b*, and *Fat1*) derived from the mouse B16-F10 melanoma cell line [89]. The authors tested different titers of the recombinant bacteriophages in immunization protocols in vivo, deriving the dose with optimal induction of specific anti-peptide antibodies. After these preliminary experiments, they deeply characterized the B-cell response induced by the different vaccines, observing a decrease in relative diversity, paralleled by expansion of clones with specific reactivity to B16-F10 cells. Therefore, this approach might be implemented to identify tumor-reactive antibodies that can then be produced by recombinant technologies and employed in immunotherapy applications.

## 7. Conclusions

Bacteriophages are promising nanocarriers for targeted delivery of therapeutic and diagnostic anticancer agents, as well as for the design of innovative vaccines. Phage-based cancer therapeutics are still in their early phase of conception, even though the evidence thus far shows promising results. Through phage display, bacteriophages can be ligand-targeted to a specific cell and their capsid can be chemically conjugated with drug molecules, in ratios that are easily ruled out. Retargeting of phages to the hallmarks of cancer has the potential to interfere with processes that sustain tumor growth and spread. The small size and variable shape (filamentous versus icosahedral) of bacteriophages allow different features. Filamentous phages, for example, have the ability to home to blood vessels, extravasate, and reach their targets when injected in vivo [90]. Compared to eukaryotic viral vectors for gene therapy, phages are non-mutant organisms whose genetic information can be easily manipulated to provide curative DNAs and RNAs. As vaccine carriers and adjuvants, they have the added advantage of easy handling and good stability. For example, to improve their safety (especially for the environment), peptide-displaying filamentous bacteriophages can be exposed to UV to lose infectivity, while keeping their immunogenicity [91]. In addition, they are resistant to degradation and retain specific binding after >6 months at room temperature, >6 weeks at 63 °C, and several days at 76 °C [92]. The same is true for DNA vaccines based on the λ phage, which are stable in the pH interval 3–11, resist >1 month at room temperature, and can be indefinitely stored at 4 °C or –70 °C [93]. Studies that specifically address the biodistribution of phages are numerous but contradictory. However, it is well-known that when administered systemically, phages reach multiple organs (skeletal muscle, heart, thymus, bone marrow, kidneys, bladder, airways, gastrointestinal tract, salivary glands, brain) in minutes and are specifically accumulated for days by others (spleen, liver, lymph nodes) whose function is to clear them out of the organism [94]. Alternative routes of administration (inhalation, oral), were also taken into consideration but their efficacy is still under debate.

In summary, the advantages of bacteriophages over other nanocarriers lie in the ease of handling, possibility of exposing variable numbers of peptides or antibodies, technical simplicity of preparation and purification, and cheapness of large-scale production. Furthermore, pages are considered safe as they have a negligible tropism for mammalian cells and a low toxicity profile even for the gut microbiome. Indeed, different phages were already approved for use in human therapy [95]. These features render bacteriophages suitable for large preparations to be distributed even in underdeveloped countries, and make them an ideal platform to implement patient-tailored applications.

## Figures and Tables

**Table 1:** Platforms for phage display.

Bacteriophage	Family	Genome	Coat Protein	Copies	Ref.
M13	*Inoviridae*	circular ssDNA	gp3 gp8	5 ~2700	[21]
λ	*Siphovirudae*	linear dsDNA	gpD, gpE gpV	405 192	[27]
MS2	*Lseviviridae*	linear ssDNA	CP	178	[28]
T4	*Myoviridae*	linear dsDNA	Hoc Soc	160 960	[29]
T7	*Podoviridae*	linear dsDNA	gp10B	415	[30]

## References

[1] Lammers T., Rizzo L.Y., Storm G., Kiessling F. (2012). Personalized nanomedicine. Clin. Cancer Res..

[2] Perik P.J., Lub-De Hooge M.N., Gietema J.A., van der Graaf W.T., de Korte M.A., Jonkman S., Kosterink J.G., van Veldhuisen D.J., Sleijfer D.T., Jager P.L. (2006). Indium-111-labeled trastuzumab scintigraphy in patients with human epidermal growth factor receptor 2-positive metastatic breast cancer. J. Clin. Oncol..

[3] Amin S., Bathe O.F. (2016). Response biomarkers: Re-envisioning the approach to tailoring drug therapy for cancer. BMC Cancer.

[4] Hait W.N., Hambley T.W. (2009). Targeted cancer therapeutics. Cancer Res..

[5] Ju Z., Sun W. (2017). Drug delivery vectors based on filamentous bacteriophages and phage-mimetic nanoparticles. Drug Deliv..

[6] Yao V.J., D’Angelo S., Butler K.S., Theron C., Smith T.L., Marchio S., Gelovani J.G., Sidman R.L., Dobroff A.S., Brinker C.J. (2016). Ligand-targeted theranostic nanomedicines against cancer. J. Control Release.

[7] Awasthi R., Roseblade A., Hansbro P.M., Rathbone M.J., Dua K., Bebawy M. (2018). Nanoparticles in cancer treatment: Opportunities and obstacles. Curr. Drug Targets.

[8] Maeda H., Wu J., Sawa T., Matsumura Y., Hori K. (2000). Tumor vascular permeability and the EPR effect in macromolecular therapeutics: A review. J. Control Release.

[9] Thomas O.S., Weber W. (2019). Overcoming physiological barriers to nanoparticle delivery-are we there yet?. Front. Bioeng. Biotechnol..

[10] Boraschi D., Italiani P., Palomba R., Decuzzi P., Duschl A., Fadeel B., Moghimi S.M. (2017). Nanoparticles and innate immunity: New perspectives on host defence. Semin. Immunol..

[11] Durymanov M.O., Rosenkranz A.A., Sobolev A.S. (2015). Current approaches for improving intratumoral accumulation and distribution of nanomedicines. Theranostics.

[12] Karimi M., Mirshekari H., Moosavi Basri S.M., Bahrami S., Moghoofei M., Hamblin M.R. (2016). Bacteriophages and phage-inspired nanocarriers for targeted delivery of therapeutic cargos. Adv. Drug Deliv. Rev..

[13] Loh B., Kuhn A., Leptihn S. (2019). The fascinating biology behind phage display: Filamentous phage assembly. Mol. Microbiol..

[14] Rakonjac J., Bennett N.J., Spagnuolo J., Gagic D., Russel M. (2011). Filamentous bacteriophage: Biology, phage display and nanotechnology applications. Curr. Issues Mol. Biol..

[15] Harada L.K., Silva E.C., Campos W.F., Del Fiol F.S., Vila M., Dabrowska K., Krylov V.N., Balcao V.M. (2018). Biotechnological applications of bacteriophages: State of the art. Microbiol. Res..

[16] Adhya S., Merril C.R., Biswas B. (2014). Therapeutic and prophylactic applications of bacteriophage components in modern medicine. Cold Spring Harb. Perspect. Med..

[17] Gamkrelidze M., Dabrowska K. (2014). T4 bacteriophage as a phage display platform. Arch. Microbiol..

[18] Lindner T., Kolmar H., Haberkorn U., Mier W. (2011). DNA libraries for the construction of phage libraries: Statistical and structural requirements and synthetic methods. Molecules.

[19] Yacoby I., Benhar I. (2008). Targeted filamentous bacteriophages as therapeutic agents. Expert. Opin. Drug Deliv..

[20] Mimmi S., Maisano D., Quinto I., Iaccino E. (2019). Phage Display: An Overview in Context to Drug Discovery. Trends Pharmacol. Sci..

[21] Saw P.E., Song E.W. (2019). Phage display screening of therapeutic peptide for cancer targeting and therapy. Protein Cell.

[22] Fagbohun O.A., Kazmierczak R.A., Petrenko V.A., Eisenstark A. (2013). Metastatic prostate cancer cell-specific phage-like particles as a targeted gene-delivery system. J. Nanobiotechnol..

[23] Davies J., Riechmann L. (1995). Antibody VH domains as small recognition units. Biotechnology.

[24] Peabody D.S. (1997). Subunit fusion confers tolerance to peptide insertions in a virus coat protein. Arch. Biochem. Biophys..

[25] Ren Z., Black L.W. (1998). Phage T4 SOC and HOC display of biologically active, full-length proteins on the viral capsid. Gene.

[26] Houshmand H., Froman G., Magnusson G. (1999). Use of bacteriophage T7 displayed peptides for determination of monoclonal antibody specificity and biosensor analysis of the binding reaction. Anal. Biochem..

[27] Casjens S.R., Hendrix R.W. (1974). Locations and amounts of major structural proteins in bacteriophage lambda. J. Mol. Biol..

[28] Koning R.I., Gomez-Blanco J., Akopjana I., Vargas J., Kazaks A., Tars K., Carazo J.M., Koster A.J. (2016). Asymmetric cryo-EM reconstruction of phage MS2 reveals genome structure in situ. Nat. Commun..

[29] Jiang J., Abu-Shilbayeh L., Rao V.B. (1997). Display of a PorA peptide from Neisseria meningitidis on the bacteriophage T4 capsid surface. Infect. Immun..

[30] Ionel A., Velazquez-Muriel J.A., Luque D., Cuervo A., Caston J.R., Valpuesta J.M., Martin-Benito J., Carrascosa J.L. (2011). Molecular rearrangements involved in the capsid shell maturation of bacteriophage T7. J. Biol. Chem..

[31] Poul M.A., Becerril B., Nielsen U.B., Morisson P., Marks J.D. (2000). Selection of tumor-specific internalizing human antibodies from phage libraries. J. Mol. Biol..

[32] Tordsson J., Lavasani S., Ohlsson L., Karlstrom P., Svedberg H., Abrahmsen L., Brodin T. (2000). A3—A novel colon and pancreatic cancer reactive antibody from a primate phage library selected using intact tumour cells. Int. J. Cancer.

[33] Pavoni E., Vaccaro P., Pucci A., Monteriu G., Beghetto E., Barca S., Dupuis M.L., De Pasquale Ceratti A., Lugini A., Cianfriglia M. (2004). Identification of a panel of tumor-associated antigens from breast carcinoma cell lines, solid tumors and testis cDNA libraries displayed on lambda phage. BMC Cancer.

[34] Larsen S.A., Meldgaard T., Fridriksdottir A.J., Lykkemark S., Poulsen P.C., Overgaard L.F., Petersen H.B., Petersen O.W., Kristensen P. (2016). Raising an antibody specific to breast cancer subpopulations using phage display on tissue sections. Cancer Genom. Proteom..

[35] Mandrup O.A., Friis N.A., Lykkemark S., Just J., Kristensen P. (2013). A novel heavy domain antibody library with functionally optimized complementarity determining regions. PLoS ONE.

[36] Mueller J., Gaertner F.C., Blechert B., Janssen K.P., Essler M. (2009). Targeting of tumor blood vessels: A phage-displayed tumor-homing peptide specifically binds to matrix metalloproteinase-2-processed collagen IV and blocks angiogenesis in vivo. Mol. Cancer Res..

[37] Zhang D., Jia H., Li W., Hou Y., Lu S., He S. (2016). Screening and identification of a phage display derived peptide that specifically binds to the CD44 protein region encoded by variable exons. J. Biomol. Screen..

[38] Zuo S., Dai G., Wang L., Wen Y., Huang Z., Yang W., Ma W., Ren X. (2019). Suppression of angiogenesis and tumor growth by recombinant T4 phages displaying extracellular domain of vascular endothelial growth factor receptor 2. Arch. Virol..

[39] Ghosh D., Peng X., Leal J., Mohanty R. (2018). Peptides as drug delivery vehicles across biological barriers. J. Pharm. Investig..

[40] Shadidi M., Sioud M. (2003). Identification of novel carrier peptides for the specific delivery of therapeutics into cancer cells. FASEB J..

[41] Bolhassani A. (2011). Potential efficacy of cell-penetrating peptides for nucleic acid and drug delivery in cancer. Biochim. Biophys. Acta.

[42] Du B., Han H., Wang Z., Kuang L., Wang L., Yu L., Wu M., Zhou Z., Qian M. (2010). Targeted drug delivery to hepatocarcinoma in vivo by phage-displayed specific binding peptide. Mol. Cancer Res..

[43] Fukuta T., Asai T., Kiyokawa Y., Nakada T., Bessyo-Hirashima K., Fukaya N., Hyodo K., Takase K., Kikuchi H., Oku N. (2017). Targeted delivery of anticancer drugs to tumor vessels by use of liposomes modified with a peptide identified by phage biopanning with human endothelial progenitor cells. Int. J. Pharm..

[44] Wang T., D’Souza G.G., Bedi D., Fagbohun O.A., Potturi L.P., Papahadjopoulos-Sternberg B., Petrenko V.A., Torchilin V.P. (2010). Enhanced binding and killing of target tumor cells by drug-loaded liposomes modified with tumor-specific phage fusion coat protein. Nanomedicine.

[45] Wang T., Hartner W.C., Gillespie J.W., Praveen K.P., Yang S., Mei L.A., Petrenko V.A., Torchilin V.P. (2014). Enhanced tumor delivery and antitumor activity in vivo of liposomal doxorubicin modified with MCF-7-specific phage fusion protein. Nanomedicine.

[46] Cai X.M., Xie H.L., Liu M.Z., Zha X.L. (2008). Inhibition of cell growth and invasion by epidermal growth factor-targeted phagemid particles carrying siRNA against focal adhesion kinase in the presence of hydroxycamptothecin. BMC Biotechnol..

[47] Huang R.K., Steinmetz N.F., Fu C.Y., Manchester M., Johnson J.E. (2011). Transferrin-mediated targeting of bacteriophage HK97 nanoparticles into tumor cells. Nanomedicine.

[48] Yi H., Ghosh D., Ham M.H., Qi J., Barone P.W., Strano M.S., Belcher A.M. (2012). M13 phage-functionalized single-walled carbon nanotubes as nanoprobes for second near-infrared window fluorescence imaging of targeted tumors. Nano Lett..

[49] Cohen B.A., Bergkvist M. (2013). Targeted in vitro photodynamic therapy via aptamer-labeled, porphyrin-loaded virus capsids. J. Photochem. Photobiol. B.

[50] Gandra N., Abbineni G., Qu X., Huai Y., Wang L., Mao C. (2013). Bacteriophage bionanowire as a carrier for both cancer-targeting peptides and photosensitizers and its use in selective cancer cell killing by photodynamic therapy. Small.

[51] Sánchez-Sánchez L., Tapia-Moreno A., Juarez-Moreno K., Patterson D.P., Cadena-Nava R.D., Douglas T., Vazquez-Duhalt R. (2015). Design of a VLP-nanovehicle for CYP450 enzymatic activity delivery. J. Nanobiotechnol..

[52] Hajitou A., Trepel M., Lilley C.E., Soghomonyan S., Alauddin M.M., Marini F.C., Restel B.H., Ozawa M.G., Moya C.A., Rangel R. (2006). A hybrid vector for ligand-directed tumor targeting and molecular imaging. Cell.

[53] Hajitou A., Lev D.C., Hannay J.A., Korchin B., Staquicini F.I., Soghomonyan S., Alauddin M.M., Benjamin R.S., Pollock R.E., Gelovani J.G. (2008). A preclinical model for predicting drug response in soft-tissue sarcoma with targeted AAVP molecular imaging. Proc. Natl. Acad. Sci. USA.

[54] Kia A., Przystal J.M., Nianiaris N., Mazarakis N.D., Mintz P.J., Hajitou A. (2012). Dual systemic tumor targeting with ligand-directed phage and Grp78 promoter induces tumor regression. Mol. Cancer Ther..

[55] Qiu Y., Peng G.L., Liu Q.C., Li F.L., Zou X.S., He J.X. (2012). Selective killing of lung cancer cells using carcinoembryonic antigen promoter and double suicide genes, thymidine kinase and cytosine deaminase (pCEA-TK/CD). Cancer Lett..

[56] Rama A.R., Hernandez R., Perazzoli G., Burgos M., Melguizo C., Velez C., Prados J. (2015). Specific colon cancer cell cytotoxicity induced by bacteriophage E gene expression under transcriptional control of carcinoembryonic antigen promoter. Int. J. Mol. Sci..

[57] Danda R., Krishnan G., Ganapathy K., Krishnan U.M., Vikas K., Elchuri S., Chatterjee N., Krishnakumar S. (2013). Targeted expression of suicide gene by tissue-specific promoter and microRNA regulation for cancer gene therapy. PLoS ONE.

[58] Higashi K., Hazama S., Araki A., Yoshimura K., Iizuka N., Yoshino S., Noma T., Oka M. (2014). A novel cancer vaccine strategy with combined IL-18 and HSV-TK gene therapy driven by the hTERT promoter in a murine colorectal cancer model. Int. J. Oncol..

[59] Dobroff A.S., D’Angelo S., Eckhardt B.L., Ferrara F., Staquicini D.I., Cardo-Vila M., Staquicini F.I., Nunes D.N., Kim K., Driessen W.H.P. (2016). Towards a transcriptome-based theranostic platform for unfavorable breast cancer phenotypes. Proc. Natl. Acad. Sci. USA.

[60] Smith T.L., Yuan Z., Cardo-Vila M., Sanchez Claros C., Adem A., Cui M.H., Branch C.A., Gelovani J.G., Libutti S.K., Sidman R.L. (2016). AAVP displaying octreotide for ligand-directed therapeutic transgene delivery in neuroendocrine tumors of the pancreas. Proc. Natl. Acad. Sci. USA.

[61] Przystal J.M., Umukoro E., Stoneham C.A., Yata T., O’Neill K., Syed N., Hajitou A. (2013). Proteasome inhibition in cancer is associated with enhanced tumor targeting by the adeno-associated virus/phage. Mol. Oncol..

[62] Yata T., Lee E.L., Suwan K., Syed N., Asavarut P., Hajitou A. (2015). Modulation of extracellular matrix in cancer is associated with enhanced tumor cell targeting by bacteriophage vectors. Mol. Cancer.

[63] Chang L., Wang G., Jia T., Zhang L., Li Y., Han Y., Zhang K., Lin G., Zhang R., Li J. (2016). Armored long non-coding RNA MEG3 targeting EGFR based on recombinant MS2 bacteriophage virus-like particles against hepatocellular carcinoma. Oncotarget.

[64] Shu Y., Haque F., Shu D., Li W., Zhu Z., Kotb M., Lyubchenko Y., Guo P. (2013). Fabrication of 14 different RNA nanoparticles for specific tumor targeting without accumulation in normal organs. RNA.

[65] Zhang Y., Leonard M., Shu Y., Yang Y., Shu D., Guo P., Zhang X. (2017). Overcoming tamoxifen resistance of human breast cancer by targeted gene silencing using multifunctional pRNA nanoparticles. ACS Nano.

[66] Guo S., Tschammer N., Mohammed S., Guo P. (2005). Specific delivery of therapeutic RNAs to cancer cells via the dimerization mechanism of phi29 motor pRNA. Hum. Gene Ther..

[67] Tarapore P., Shu Y., Guo P., Ho S.M. (2011). Application of phi29 motor pRNA for targeted therapeutic delivery of siRNA silencing metallothionein-IIA and survivin in ovarian cancers. Mol. Ther..

[68] Sartorius R., Bettua C., D’Apice L., Caivano A., Trovato M., Russo D., Zanoni I., Granucci F., Mascolo D., Barba P. (2011). Vaccination with filamentous bacteriophages targeting DEC-205 induces DC maturation and potent anti-tumor T-cell responses in the absence of adjuvants. Eur. J. Immunol..

[69] Sartorius R., D’Apice L., Prisco A., De Berardinis P. (2019). Arming filamentous bacteriophage, a nature-made nanoparticle, for new vaccine and immunotherapeutic strategies. Pharmaceutics.

[70] Krut O., Bekeredjian-Ding I. (2018). Contribution of the iImmune response to phage therapy. J. Immunol..

[71] Gaubin M., Fanutti C., Mishal Z., Durrbach A., De Berardinis P., Sartorius R., Del Pozzo G., Guardiola J., Perham R.N., Piatier-Tonneau D. (2003). Processing of filamentous bacteriophage virions in antigen-presenting cells targets both HLA class I and class II peptide loading compartments. DNA Cell Biol..

[72] Sartorius R., Pisu P., D’Apice L., Pizzella L., Romano C., Cortese G., Giorgini A., Santoni A., Velotti F., De Berardinis P. (2008). The use of filamentous bacteriophage fd to deliver MAGE-A10 or MAGE-A3 HLA-A2-restricted peptides and to induce strong antitumor CTL responses. J. Immunol..

[73] Van Houten N.E., Henry K.A., Smith G.P., Scott J.K. (2010). Engineering filamentous phage carriers to improve focusing of antibody responses against peptides. Vaccine.

[74] Van Houten N.E., Zwick M.B., Menendez A., Scott J.K. (2006). Filamentous phage as an immunogenic carrier to elicit focused antibody responses against a synthetic peptide. Vaccine.

[75] Cuesta A.M., Suarez E., Larsen M., Jensen K.B., Sanz L., Compte M., Kristensen P., Alvarez-Vallina L. (2006). Enhancement of DNA vaccine potency through linkage of antigen to filamentous bacteriophage coat protein III domain I. Immunology.

[76] Bartolacci C., Andreani C., Curcio C., Occhipinti S., Massaccesi L., Giovarelli M., Galeazzi R., Iezzi M., Tilio M., Gambini V. (2018). Phage-based anti-HER2 vaccination can circumvent immune tolerance against breast cancer. Cancer Immunol. Res..

[77] Roehnisch T., Then C., Nagel W., Blumenthal C., Braciak T., Donzeau M., Bohm T., Bourquin C., Oduncu F. (2013). Chemically linked phage idiotype vaccination in the murine B cell lymphoma 1 model. J. Transl. Med..

[78] Clark J.R., March J.B. (2004). Bacteriophage-mediated nucleic acid immunisation. FEMS Immunol. Med. Microbiol..

[79] March J.B., Clark J.R., Jepson C.D. (2004). Genetic immunisation against hepatitis B using whole bacteriophage lambda particles. Vaccine.

[80] Ghaemi A., Soleimanjahi H., Gill P., Hassan Z., Jahromi S.R., Roohvand F. (2010). Recombinant lambda-phage nanobioparticles for tumor therapy in mice models. Genet. Vaccines Ther..

[81] Ghaemi A., Soleimanjahi H., Gill P., Hassan Z.M., Razeghi S., Fazeli M., Razavinikoo S.M. (2011). Protection of mice by a lambda-based therapeutic vaccine against cancer associated with human papillomavirus type 16. Intervirology.

[82] Thomas B.S., Nishikawa S., Ito K., Chopra P., Sharma N., Evans D.H., Tyrrell D.L., Bathe O.F., Rancourt D.E. (2012). Peptide vaccination is superior to genetic vaccination using a recombineered bacteriophage lambda subunit vaccine. Vaccine.

[83] Arab A., Nicastro J., Slavcev R., Razazan A., Barati N., Nikpoor A.R., Brojeni A.A.M., Mosaffa F., Badiee A., Jaafari M.R. (2018). Lambda phage nanoparticles displaying HER2-derived E75 peptide induce effective E75-CD8(+) T response. Immunol. Res..

[84] Barati N., Razazan A., Nicastro J., Slavcev R., Arab A., Mosaffa F., Nikpoor A.R., Badiee A., Jaafari M.R., Behravan J. (2018). Immunogenicity and antitumor activity of the superlytic lambdaF7 phage nanoparticles displaying a HER2/neu-derived peptide AE37 in a tumor model of BALB/c mice. Cancer Lett..

[85] Razazan A., Nicastro J., Slavcev R., Barati N., Arab A., Mosaffa F., Jaafari M.R., Behravan J. (2019). Lambda bacteriophage nanoparticles displaying GP2, a HER2/neu derived peptide, induce prophylactic and therapeutic activities against TUBO tumor model in mice. Sci. Rep..

[86] Ren S.X., Ren Z.J., Zhao M.Y., Wang X.B., Zuo S.G., Yu F. (2009). Antitumor activity of endogenous mFlt4 displayed on a T4 phage nanoparticle surface. Acta Pharmacol. Sin..

[87] Ren S., FengYu, Zuo S., Zhao M., Wang X., Wang X., Chen Y., Wu Z., Ren Z. (2011). Inhibition of tumor angiogenesis in lung cancer by T4 phage surface displaying mVEGFR2 vaccine. Vaccine.

[88] Pouyanfard S., Bamdad T., Hashemi H., Bandehpour M., Kazemi B. (2012). Induction of protective anti-CTL epitope responses against HER-2-positive breast cancer based on multivalent T7 phage nanoparticles. PLoS ONE.

[89] Shukla G.S., Sun Y.J., Pero S.C., Sholler G.S., Krag D.N. (2018). Immunization with tumor neoantigens displayed on T7 phage nanoparticles elicits plasma antibody and vaccine-draining lymph node B cell responses. J. Immunol. Methods..

[90] Bakhshinejad B., Karimi M., Khalaj-Kondori M. (2015). Phage display: Development of nanocarriers for targeted drug delivery to the brain. Neural. Regen. Res..

[91] Samoylova T.I., Norris M.D., Samoylov A.M., Cochran A.M., Wolfe K.G., Petrenko V.A., Cox N.R. (2012). Infective and inactivated filamentous phage as carriers for immunogenic peptides. J. Virol. Methods.

[92] Brigati J.R., Petrenko V.A. (2005). Thermostability of landscape phage probes. Anal. Bioanal. Chem..

[93] Jepson C.D., March J.B. (2004). Bacteriophage lambda is a highly stable DNA vaccine delivery vehicle. Vaccine.

[94] Dufour N., Delattre R., Debarbieux L. (2018). In Vivo Bacteriophage Biodistribution. Methods Mol. Biol..

[95] Rehman S., Ali Z., Khan M., Bostan N., Naseem S. (2019). The dawn of phage therapy. Rev. Med. Virol..

